# Supplementary material for: Using TLC-MALDI-TOF to Interrogate In Vitro Peptidyl Proximal Preferences of PARP14 and Glycohydrolase Specificity
Source: Molecules. 2023 Aug 15;28(16):6061. doi: 10.3390/molecules28166061 (PMC10459978; doi:10.3390/molecules28166061)
Supplement: Supplementary file 1 [file molecules-28-06061-s001.zip › molecules-2555214-supplementary.pdf]

Supporting Information

**Peptide**	**Peptide Sequence**	**Unmodified m/z**	**+MAR m/z**	**pI**
P14p1	SLNYKSTSSGHR**E**ISSPR	2006.16	2546.16	10.49
P14p2	WHR**E**ISSPR	1166.59	1706.59	10.95
P14p3	SLNYKSTSSGHR	1336.43	1876.43	10.50
P14p4	RWSSGHR**E**ISS	1301.38	1841.38	10.95
P14p5	SLNYKSTSSGHR**E**IS	1664.81	2204.81	9.67
P14p6	HR**E**ISW	826.91	1366.91	7.90
P14p7	HR**D**ISW	812.88	1352.88	6.74
P14p8	AR**E**ISWH	897.99	1437.99	7.90
P14p9	HA**E**ISWR	897.99	1437.99	7.90
P14p10	HR**A**ISWR	925.06	1465.06	12.00
P14p11	HR**E**ASW	784.83	1324.83	7.90
P14p12	HR**E**IAW	810.91	1350.91	7.90
P14p13	HM**E**ISWR	958.11	1498.11	7.90
P14p14	HK**E**ISW	798.90	1338.90	7.88
P14p15	HIR**E**SW	826.91	1366.91	7.90
P14p16	HISR**E**W	826.91	1366.91	7.90

Table S1. Peptides analyzed in the current study. Peptide sequence, unmodified *m/z*, expected +MAR *m/z*, and isoelectric point (pI) are indicated.

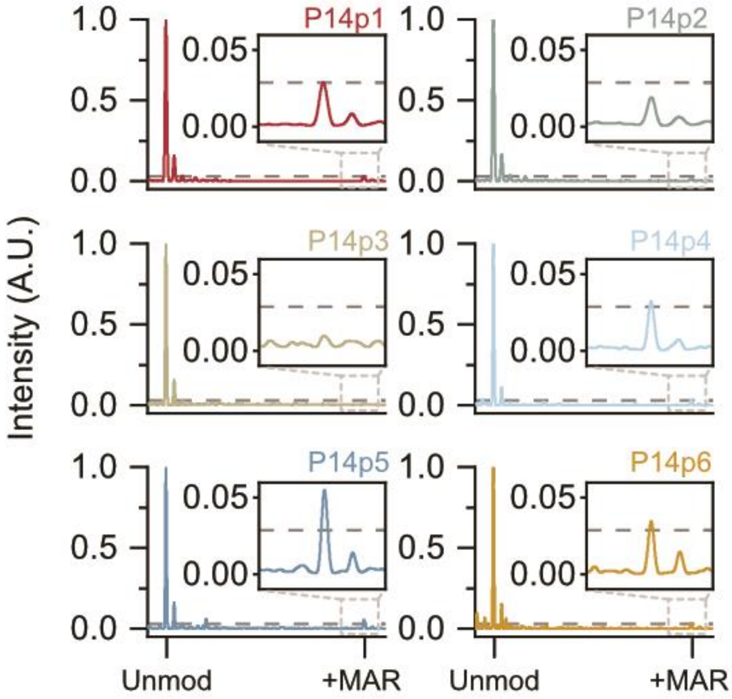

Figure S1. ADP-ribosylation of P14c selective peptides by P14-∆553. P14-∆553 and the indicated peptides were incubated in the presence of NAD^+ and subjected to TLC-MALDI to visualize the resulting increase in *m/z* due to MAR (+541 Da). The dashed line represents the intensity observed for ADP-ribosylation of P14p1 by P14-∆553 and the inset highlights the +MAR spectra.

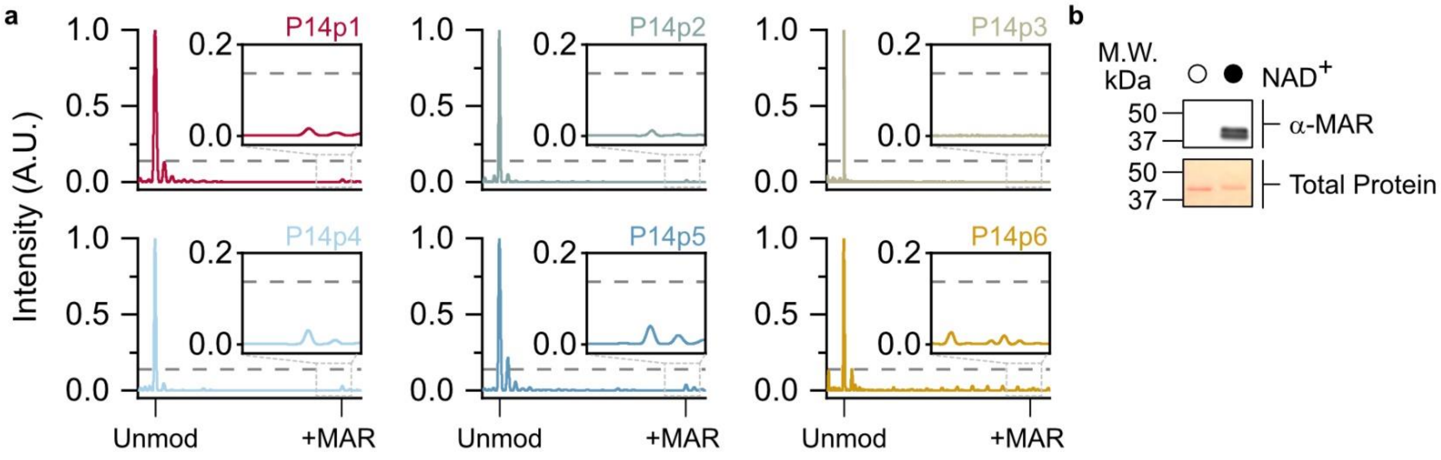

Figure S2. Minimal ADP-ribosylation of P14 selective peptides by P15. (a) P15 and the indicated peptides were incubated in the presence of NAD^+ and subjected to TLC-MALDI to visualize the resulting increase in *m/z* due to MAR (+541 Da). The dashed line represents the intensity observed for ADP-ribosylation of P14p6 by P14c and the inset highlights the +MAR spectra. (b) *In vitro* P15 ADP-ribosylation assay.

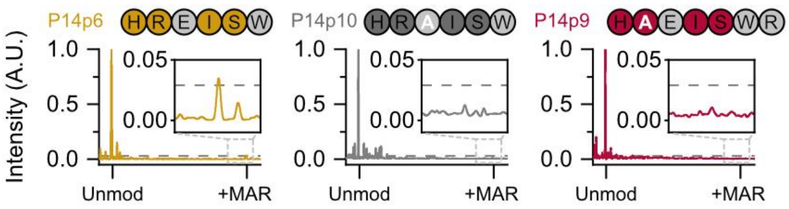

Figure S3. ADP-ribosylation of key alanine mutants by P14-∆553. P14-∆553 and the indicated peptides were incubated in the presence of NAD⁺ and subjected to TLC-MALDI to visualize the resulting increase in *m/z* due to MAR (+541 Da). The dashed line represents the intensity observed for ADP-ribosylation of P14p6 by P14-∆553 and the inset highlights the +MAR spectra.

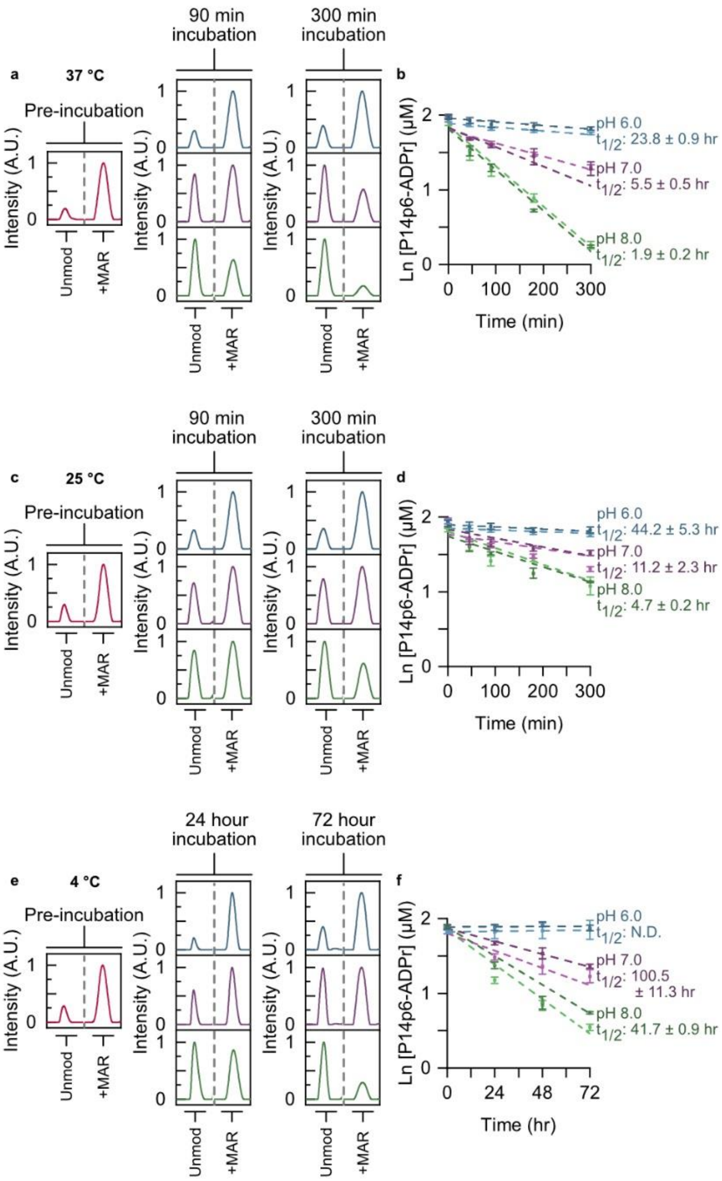

Figure S4. Non-enzymatic hydrolysis of MAR is sequence independent. (a) Synthesized MAR—P14p9 peptide was equilibrated at 37 °C and pH 6.0 (blue), pH 7.0 (purple), or pH 8.0 (green) and MAR hydrolysis was monitored at the indicated times using TLC-MALDI. The unmodified and modified peaks are shown for comparison. (b) MS spectra were integrated to determine the relative levels of MAR hydrolysis and fit to a pseudo first-order rate expression to determine the half-life of the MAR modification (mean ± S.E.M., *n* = 3). The fits obtained for P14p6 are shown for comparison (lighter coloring). (c) Experiments were performed as in (a) at 25 °C. (d) Determination of MAR half-lives at 25 °C. (e) Experiments were performed as in (a) at 4 °C. (f) Determination of MAR half-lives at 4 °C.

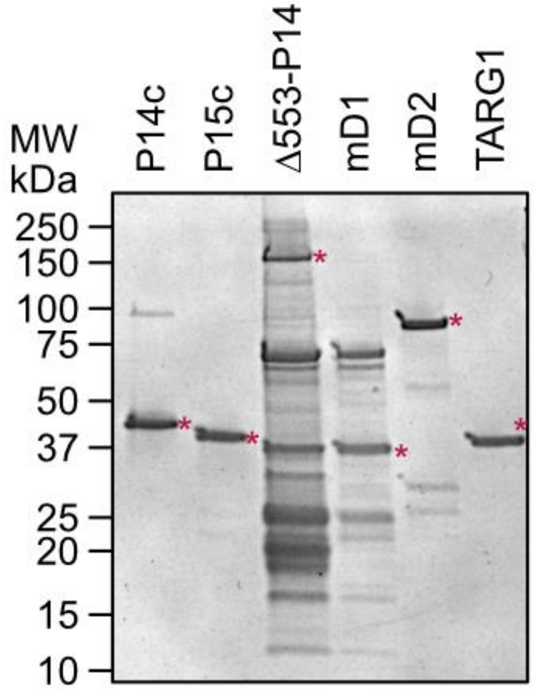

Figure S5. Purified proteins utilized in this study. All proteins were loaded at a concentration of 500 µM and visualized using Coomassie stain following separation on an SDS-PAGE gel. The expected recombinant protein for each sample is indicated by an *.
